# 5-Phenyl-3-(2-phosphono­eth­yl)-1,2,3-triazol-1-ium chloride

**DOI:** 10.1107/S2414314622001894

**Published:** 2022-02-25

**Authors:** Elpiniki Chachlaki, Duane Choquesillo-Lazarte, Konstantinos D. Demadis

**Affiliations:** a Crystal Engineering, Growth and Design Laboratory, Department of Chemistry, University of Crete, Voutes Campus, Crete, GR-71003, Greece; b Laboratorio de Estudios Cristalográficos, IACT, CSIC-Universidad de Granada, Granada-18100, Spain; University of Neuchâtel, Switzerland

**Keywords:** phospho­nate, triazole, hydrogen bonding, click chemistry, crystal structure

## Abstract

The new triazole-functionalized phospho­nic acid 5-phenyl-3-(2-phosphono­eth­yl)-1,2,3-triazol-1-ium chloride, C_10_H_13_N_3_O_3_P^+^·Cl^−^ (PTEPHCl), was synthesized by the ‘click’ reaction of the alkyl azide diethyl-(2-azido­eth­yl)phospho­nate with phenyl­acetyl­ene to give the dieth­yl[2-(4-phenyl-1*H*-1,2,3-triazol-1-yl)eth­yl]phospho­nate ester, which was then hydrolyzed under acidic conditions (HCl) to give the ‘free’ phospho­nic acid. The use of HCl for the hydrolysis caused protonation of the triazole ring, rendering the compound cationic, charged-balanced by a Cl^−^ anion. There are extensive hydrogen-bonding inter­actions in the structure of PTEPHCl, involving the phospho­nic acid (–PO_3_H_2_) group, the triazolium ring and the Cl^−^ anion.

## Abstract 2

This new triazole-functionalized phospho­nic acid, PTEPHCl, was synthesized by the ‘click’ reaction of the alkyl azide diethyl-(2-azido­eth­yl)phospho­nate with phenyl­acetyl­ene to give the dieth­yl[2-(4-phenyl-1*H*-1,2,3-triazol-1-yl)eth­yl]phospho­nate ester, which was then hydrolyzed under acidic conditions (HCl) to give the ‘free’ phospho­nic acid. The use of HCl for the hydrolysis caused protonation of the triazole ring, rendering the compound cationic, charged-balanced by a Cl^−^ anion.

## Graphical abstract

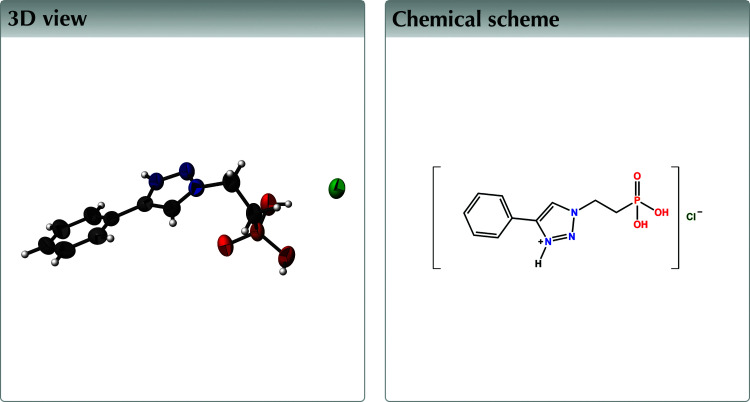

## Structure description

The exponential growth of the field of MOFs and coordination polymers over the past few decades is partially due to the design, synthesis and functionalization of appropriate linkers (Zaręba, 2017 ▸). Although the field was initiated with compounds that were mainly based on polycarboxyl­ate linkers, its continuous development currently embraces virtually all mol­ecules that are able to bind to metals. Among the plethora of ligands, (poly)phospho­nic acids stand out because they can construct networks with high thermal and hydrolytic stability (Clearfield & Demadis, 2012 ▸). The field of metal phospho­nates also relies on the availability of proper phospho­nate linkers that offer structural diversity and can produce metal phospho­nate compounds with attractive properties. Most of the published work on new phospho­nic acids is based on two synthetic methodologies: (i) the Arbuzov reaction (Babu *et al.*, 2017 ▸) and (ii) the Mannich-type (a.k.a. Moedritzer–Irani) reaction (Villemin *et al.*, 2021 ▸). The Arbuzov reaction can convert an organic halide to a phospho­nic acid group, whereas Mannich-type reactions transform an amine group to an amino­methyl­ene­phospho­nic group. Both synthetic strategies aim at introducing a phospho­nic acid moiety to a pre-formed organic fragment. We recently initiated synthetic efforts that are based on ‘click’ chemistry. Specifically, the approach is based on a ‘reactive’ organic mol­ecule that already contains a phospho­nic acid group, but can undergo other transformations elsewhere on the backbone.

The reaction of an organic azide with an alkyne to give a triazole is a well-known process (Mukherjee *et al.*, 2013 ▸). Herein, this transformation was performed on an organic azide that already contains a phospho­nate group on its backbone to yield a phospho­nate-modified triazole. Specifically, an alkyl azide [diethyl-(2-azido­eth­yl)phospho­nate] was reacted with an aromatic alkyne (phenyl­acetyl­ene) to give dieth­yl[2-(4-phenyl-1*H*-1,2,3-triazol-1-yl)eth­yl]phospho­nate ester. This ester was then hydrolyzed in acidic conditions to give 5-phenyl-3-(2-phosphono­eth­yl)-1,2,3-triazol-1-ium chloride (PTEPHCl). In the present work, we report the crystal structure of the above-mentioned triazole-functional­ized phospho­nic acid PTEPHCl.

**Molecular structure**

**Figure 1:**
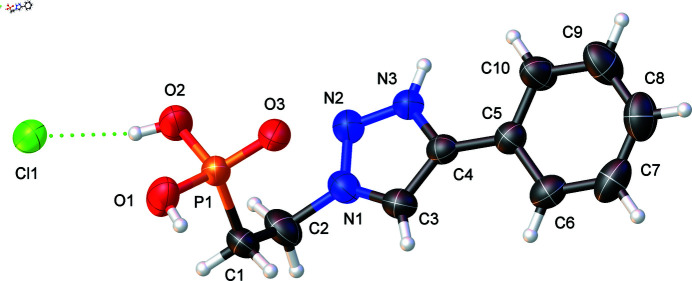
Mol­ecular structure of 5-phenyl-3-(2-phosphono­eth­yl)-1,2,3-triazol-1-ium chloride with the atom-labeling scheme. Displacement ellipsoids are shown at the 50% probability level. Color code: P orange, O red, C black, N blue, Cl green, H white.

Fig. 1 ▸ shows the mol­ecular structure of 5-phenyl-3-(2-phosphono­eth­yl)-1,2,3-triazol-1-ium chloride. Because HCl was used for the ester hydrolysis, the N3 atom of the triazole ring and the O1 and O2 atoms of the phospho­nate group are protonated due to the synthesis of PTEPHCl at low pH, hence a chloride counter-ion (Cl1) is found in the structure.

There are two ‘long’ P—O bonds [P1—O1 = 1.5526 (16) and P1—O2 = 1.5513 (16) Å] and one ‘short’ P—O bond [P1—O3 = 1.4805 (14) Å]. The ‘long’ P—O bonds correspond to the P—O—H moieties and the ‘short’ P—O bond corresponds to the phosphoryl P=O moiety. All P—O bond lengths have the expected values (Colodrero *et al.*, 2013 ▸). The bond lengths of the triazolium moiety [N1—N2 = 1.317 (2) Å, N1—C2 = 1.474 (3) Å, N1—C3 = 1.346 (3) Å, N2—N3 = 1.318 (2) Å, N3—C4 = 1.352 (2) Å] are very similar to those in 1,2,4-triazolium chloride (Bujak & Zaleski, 2002 ▸).

**Hydrogen bonding**

**Figure 2:**
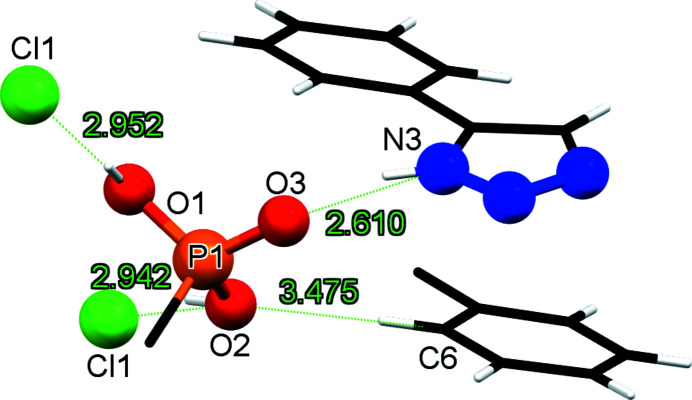
Hydrogen-bonding scheme of the phospho­nic acid group in the structure of PTEPHCl.

The phospho­nic acid moiety forms four hydrogen-bonding inter­actions (Fig. 2 ▸ and Table 1 ▸). Specifically, each of the two P—O—H groups inter­acts with a different Cl^−^ counter-ion, with contacts O1⋯Cl1 = 2.9521 (16) Å and O2⋯Cl1 = 2.9422 (17) Å. The phosphoryl P=O group forms a hydrogen bond with the N—H portion of the triazolium ring [O3⋯N3 2.610 (2) Å]. Finally, the benzene ring inter­acts with a phospho­nate oxygen through a weak C—H⋯O contact at 3.476 (3) Å (C6⋯O2).

**π–π stacking inter­actions**

There is only one type of very weak π–π stacking inter­action in the structure of 5-phenyl-3-(2-phosphono­eth­yl)-1,2,3-triazol-1-ium chloride. The centroid-to-centroid distance is 4.0423 (15) Å, with the rings being ‘shifted’ from one another (slippage distance between the rings: 2.222 Å) and parallel.

**Crystal packing**

**Figure 3:**
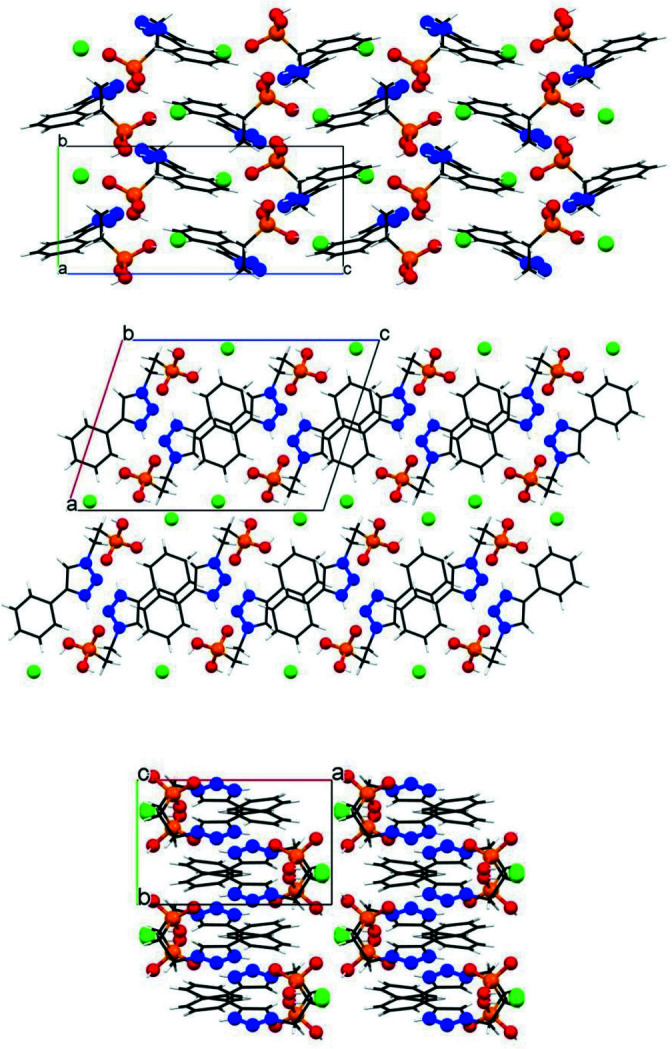
Packing of 5-phenyl-3-(2-phosphono­eth­yl)-1,2,3-triazol-1-ium chloride along the *a*- (upper), *b*- (middle), and *c*-axes (lower).

Fig. 3 ▸ shows the packing along the three axes. The π–π stacking inter­actions are parallel to the *b* axis. The chloride anions form corrugated sheets [‘short’ Cl⋯Cl distances at 4.9455 (12) Å and ‘long’ Cl⋯Cl distances at 6.4564 (9) Å] that are parallel to the *bc* plane.

## Synthesis and crystallization

**Reagents and materials**

All starting materials were obtained from commercial sources and used without further purification. The reagents diethyl 2-bromo­ethyl­phospho­nate (97%), phenyl­acetyl­ene (98+%), copper sulfate penta­hydrate (99%), zinc nitrate hexa­hydrate (98%) and ethyl­enedi­amine­tetra­acetic acid (98%) were from Alfa Aesar. Sodium azide and l-ascorbic acid were from Serva. Sodium sulfate was from Merck. Di­chloro­methane, tetra­hydro­furan (THF), hydro­chloric acid (37%) and nitric acid (70%) were from Scharlau. Ion-exchange-column deionized water was used.

**Synthesis of 5-phenyl-3-(2-phosphono­eth­yl)-1,2,3-triazol-1-ium chloride (PTEPHCl)**

Three distinct steps were followed for the syntheses of the ligand PTEP. The first step was the synthesis of diethyl-(2-azido­eth­yl)phospho­nate, following a properly adapted published procedure (Sheikhi *et al.*, 2018 ▸). Specifically, sodium azide (10.6 g, 163.05 mmol) was added to a solution of diethyl-2-bromo­ethyl­phospho­nate (10.4 g, 42.44 mmol) in water (50 mL). The reaction mixture was stirred at 338 K for 24 h. Then, extraction was carried out with di­chloro­methane (4 × 50 mL) and the organic phase was collected and dried over sodium sulfate. After filtration, a yellow oil was obtained, which is diethyl-(2-azido­eth­yl)phospho­nate. The second step included the reaction of diethyl-(2-azido­eth­yl)phospho­nate (3 mL, 2.07 mmol) with phenyl acetyl­ene (895 µL, 1.035 mmol) in THF (67.5 mL), in the presence of copper sulfate (1.198 g 0.64 mmol) and l-ascorbic acid (0.218 g, 1.24 mmol) to produce dieth­yl[2-(4-phenyl-1*H*-1,2,3-triazol-1-yl)eth­yl]phospho­nate ester. The reaction mixture was heated at 313 K under vigorous stirring for 48 h. After filtration, the filtrate was mixed with di­chloro­methane (50 mL) and an aqueous solution of the Cu^2+^ chelant ethyl­enedi­amine­tetra­acetic acid (50 mL, 0.2 *M*) and the mixture was stirred for ∼1 h. After extraction with di­chloro­methane (4 × 50 mL) and evaporation, dieth­yl[2-(4-phenyl-1*H*-1,2,3-triazol-1-yl)eth­yl]phospho­nate ester was obtained in solid form. Finally, the latter (0.5 g) was hydrolyzed with 25 mL of H_2_O and 30 mL of HCl at 373 K for 48 h, giving 5-phenyl-3-(2-phosphono­eth­yl)-1,2,3-triazol-1-ium chloride in crystalline form (yield: 0.3 g, 60%). The crystal used for the data collection was handled under inert conditions. It was manipulated while immersed in a perfluoro­polyether protecting oil and mounted on a MiTeGen Micromount™.

^1^H NMR (300 MHz, DMSO-*d*_6_) δ 8.51 (*s*, 1H), 7.93 (*d*, 2H), 7.67 (*m*, 3H), 4.82 (*m*, 2H), 2.52 (*m*, 2H). ^13^C NMR (75.5 MHz, DMSO-*d*_6_) δ 146.71, 131.27, 129.38, 128.28, 125.53, 121.87, 45.42, 30.45 (*d*, *J*_CP_ = 134.5 Hz). ^31^P NMR (121.5 MHz, DMSO-*d*_6_) δ 20.17.

## Refinement

Crystal data, data collection and structure refinement details are summarized in Table 2 ▸.

## Supplementary Material

Crystal structure: contains datablock(s) I. DOI: 10.1107/S2414314622001894/tx4001sup1.cif

Structure factors: contains datablock(s) I. DOI: 10.1107/S2414314622001894/tx4001Isup4.hkl

Supporting information file. DOI: 10.1107/S2414314622001894/tx4001Isup5.mol

Supporting information file. DOI: 10.1107/S2414314622001894/tx4001Isup4.cml

CCDC reference: 2145106

Additional supporting information:  crystallographic information; 3D view; checkCIF report

## Figures and Tables

**Table 1:** Hydrogen-bond geometry (Å, °)

*D*—H⋯*A*	*D*—H	H⋯*A*	*D*⋯*A*	*D*—H⋯*A*
O1—H1⋯Cl1^i^	0.82	2.15	2.9521 (16)	167
O2—H2⋯Cl1	0.82	2.16	2.9422 (17)	160
N3—H3⋯O3^ii^	0.86	1.78	2.610 (2)	162
C6—H6⋯O2^iii^	0.93	2.58	3.476 (3)	163

Symmetry codes: (i) 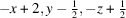 ; (ii) 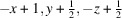 ; (iii) 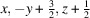 .

**Table 2:** Experimental details

Crystal data	
Chemical formula	C_10_H_13_N_3_O_3_P^+^·Cl^−^
*M*_r_	289.65
Crystal system, space group	Monoclinic, *P*2_1_/*c*
Temperature (K)	298
*a*, *b*, *c* (Å)	11.5857 (6), 7.0616 (4), 16.6118 (9)
β (°)	108.222 (2)
*V* (Å^3^)	1290.92 (12)
*Z*	4
Radiation type	Cu *K*α
μ (mm^−1^)	3.86
Crystal size (mm)	0.12 × 0.09 × 0.08

Data collection	
Diffractometer	Bruker D8 Venture
Absorption correction	Multi-scan (*SADABS*; Bruker, 2016 ▸)
*T*_min_, *T*_max_	0.524, 0.753
No. of measured, independent and observed [*I* > 2σ(*I*)] reflections	11621, 2268, 2048
*R*_int_	0.046
(sin θ/λ)_max_ (Å^−1^)	0.596

Refinement	
*R*[*F* ^2^ > 2σ(*F* ^2^)], *wR*(*F* ^2^), *S*	0.042, 0.129, 1.11
No. of reflections	2268
No. of parameters	166
H-atom treatment	H-atom parameters constrained
Δρ_max_, Δρ_min_ (e Å^−3^)	0.21, −0.34

Computer programs: *APEX3* (Bruker, 2019 ▸), *SAINT* (Bruker, 2016 ▸), *SHELXT* (Sheldrick, 2015*a* ▸), *SHELXL* (Sheldrick, 2015*b* ▸) and *OLEX2* (Dolomanov *et al.*, 2009 ▸).

## full crystallographic data

### Crystal data

**Table:**

C_10_H_13_N_3_O_3_P^+^·Cl^−^	*F*(000) = 600
*M**_r_* = 289.65	*D*_x_ = 1.490 Mg m^−^^3^
Monoclinic, *P*2_1_/*c*	Cu *K*α radiation, λ = 1.54178 Å
*a* = 11.5857 (6) Å	Cell parameters from 9685 reflections
*b* = 7.0616 (4) Å	θ = 4.0–66.7°
*c* = 16.6118 (9) Å	µ = 3.86 mm^−^^1^
β = 108.222 (2)°	*T* = 298 K
*V* = 1290.92 (12) Å^3^	Plate, colourless
*Z* = 4	0.12 × 0.09 × 0.08 mm

### Data collection

**Table:**

Bruker D8 Venture diffractometer	2048 reflections with *I* > 2σ(*I*)
φ and ω scans	*R*_int_ = 0.046
Absorption correction: multi-scan (SADABS; Bruker, 2016)	θ_max_ = 66.7°, θ_min_ = 4.0°
*T*_min_ = 0.524, *T*_max_ = 0.753	*h* = −12→13
11621 measured reflections	*k* = −8→8
2268 independent reflections	*l* = −18→19

### Refinement

**Table:**

Refinement on *F*^2^	Hydrogen site location: inferred from neighbouring sites
Least-squares matrix: full	H-atom parameters constrained
*R*[*F*^2^ > 2σ(*F*^2^)] = 0.042	*w* = 1/[σ^2^(*F*_o_^2^) + (0.0842*P*)^2^ + 0.2113*P*] where *P* = (*F*_o_^2^ + 2*F*_c_^2^)/3
*wR*(*F*^2^) = 0.129	(Δ/σ)_max_ < 0.001
*S* = 1.11	Δρ_max_ = 0.21 e Å^−^^3^
2268 reflections	Δρ_min_ = −0.34 e Å^−^^3^
166 parameters	Extinction correction: SHELXL2019/1 (Sheldrick 2015b), Fc^*^=kFc[1+0.001xFc^2^λ^3^/sin(2θ)]^-1/4^
0 restraints	Extinction coefficient: 0.0086 (14)

### Special details

**Table:**

Geometry. All esds (except the esd in the dihedral angle between two l.s. planes) are estimated using the full covariance matrix. The cell esds are taken into account individually in the estimation of esds in distances, angles and torsion angles; correlations between esds in cell parameters are only used when they are defined by crystal symmetry. An approximate (isotropic) treatment of cell esds is used for estimating esds involving l.s. planes.
Refinement. All hydrogen atoms were located in difference Fourier maps and included as fixed contributions riding on attached atoms with isotropic thermal displacement parameters 1.2 or 1.5 times those of the respective atom.

### Fractional atomic coordinates and isotropic or equivalent isotropic displacement parameters (Å^2^)

**Table:**

	*x*	*y*	*z*	*U*_iso_*/*U*_eq_	
P1	0.81725 (4)	0.61394 (8)	0.26679 (3)	0.0473 (2)	
O1	0.91887 (15)	0.4728 (3)	0.26434 (10)	0.0660 (5)	
H1	0.946479	0.421812	0.310754	0.099*	
O2	0.78145 (14)	0.7154 (3)	0.17962 (10)	0.0663 (5)	
H2	0.838534	0.711018	0.160392	0.099*	
O3	0.70907 (13)	0.5261 (2)	0.28078 (9)	0.0570 (4)	
N1	0.68960 (15)	0.9187 (2)	0.35631 (11)	0.0480 (4)	
N2	0.59278 (15)	0.9687 (3)	0.29376 (10)	0.0497 (4)	
N3	0.50156 (14)	0.9160 (2)	0.32010 (10)	0.0446 (4)	
H3	0.426572	0.931250	0.290694	0.054*	
C1	0.88515 (18)	0.7829 (3)	0.34778 (14)	0.0530 (5)	
H1A	0.902609	0.720386	0.402270	0.064*	
H1B	0.962183	0.821985	0.341559	0.064*	
C2	0.8107 (2)	0.9598 (3)	0.34921 (17)	0.0626 (6)	
H2A	0.855085	1.038465	0.396689	0.075*	
H2B	0.801074	1.031479	0.297697	0.075*	
C3	0.66201 (18)	0.8378 (3)	0.42134 (13)	0.0468 (5)	
H3A	0.716089	0.792849	0.471680	0.056*	
C4	0.53734 (17)	0.8355 (3)	0.39786 (12)	0.0406 (4)	
C5	0.45429 (18)	0.7694 (2)	0.44224 (12)	0.0419 (5)	
C6	0.4974 (2)	0.7322 (3)	0.52898 (13)	0.0509 (5)	
H6	0.579280	0.748521	0.558805	0.061*	
C7	0.4176 (3)	0.6708 (3)	0.57037 (15)	0.0634 (6)	
H7	0.446686	0.644005	0.628029	0.076*	
C8	0.2960 (3)	0.6489 (3)	0.52761 (18)	0.0678 (7)	
H8	0.242943	0.611045	0.556500	0.081*	
C9	0.2534 (2)	0.6829 (4)	0.44228 (18)	0.0727 (7)	
H9	0.171424	0.665745	0.412999	0.087*	
C10	0.3319 (2)	0.7427 (3)	0.39929 (16)	0.0573 (6)	
H10	0.302340	0.765053	0.341264	0.069*	
Cl1	0.94890 (5)	0.76568 (8)	0.07972 (3)	0.0583 (3)	

### Atomic displacement parameters (Å^2^)

**Table:**

	*U*^11^	*U*^22^	*U*^33^	*U*^12^	*U*^13^	*U*^23^
P1	0.0364 (3)	0.0693 (4)	0.0365 (4)	0.0015 (2)	0.0118 (2)	0.0013 (2)
O1	0.0610 (10)	0.0914 (12)	0.0490 (9)	0.0254 (8)	0.0223 (7)	0.0080 (8)
O2	0.0475 (9)	0.1068 (13)	0.0442 (9)	0.0135 (8)	0.0136 (7)	0.0171 (8)
O3	0.0468 (8)	0.0797 (10)	0.0457 (8)	−0.0157 (7)	0.0163 (6)	−0.0101 (7)
N1	0.0392 (9)	0.0508 (9)	0.0543 (10)	−0.0028 (7)	0.0150 (7)	−0.0041 (7)
N2	0.0458 (9)	0.0583 (10)	0.0469 (10)	0.0002 (7)	0.0174 (8)	0.0004 (7)
N3	0.0383 (8)	0.0560 (9)	0.0385 (9)	0.0008 (7)	0.0104 (6)	−0.0007 (7)
C1	0.0325 (10)	0.0742 (14)	0.0510 (12)	−0.0047 (9)	0.0110 (9)	−0.0007 (10)
C2	0.0422 (11)	0.0659 (14)	0.0816 (16)	−0.0130 (10)	0.0220 (11)	−0.0053 (12)
C3	0.0401 (10)	0.0492 (10)	0.0470 (11)	−0.0016 (8)	0.0076 (8)	−0.0018 (8)
C4	0.0419 (9)	0.0394 (9)	0.0384 (10)	0.0006 (7)	0.0097 (7)	−0.0037 (7)
C5	0.0470 (11)	0.0379 (9)	0.0414 (11)	0.0027 (7)	0.0145 (8)	−0.0006 (7)
C6	0.0610 (13)	0.0468 (11)	0.0419 (11)	0.0008 (9)	0.0118 (9)	−0.0026 (8)
C7	0.1016 (19)	0.0488 (12)	0.0472 (12)	0.0034 (12)	0.0339 (12)	0.0020 (9)
C8	0.0832 (18)	0.0559 (13)	0.0814 (18)	0.0002 (12)	0.0503 (15)	0.0100 (12)
C9	0.0525 (13)	0.0751 (16)	0.094 (2)	−0.0061 (12)	0.0289 (13)	0.0171 (14)
C10	0.0476 (12)	0.0685 (14)	0.0525 (13)	−0.0047 (9)	0.0110 (10)	0.0134 (10)
Cl1	0.0507 (4)	0.0795 (4)	0.0435 (4)	0.0062 (2)	0.0130 (3)	−0.0025 (2)

### Geometric parameters (Å, º)

**Table:**

P1—O1	1.5526 (16)	C2—H2B	0.9700
P1—O2	1.5513 (16)	C3—H3A	0.9300
P1—O3	1.4805 (14)	C3—C4	1.373 (3)
P1—C1	1.786 (2)	C4—C5	1.460 (3)
O1—H1	0.8200	C5—C6	1.394 (3)
O2—H2	0.8200	C5—C10	1.386 (3)
N1—N2	1.317 (2)	C6—H6	0.9300
N1—C2	1.474 (3)	C6—C7	1.383 (3)
N1—C3	1.346 (3)	C7—H7	0.9300
N2—N3	1.318 (2)	C7—C8	1.373 (4)
N3—H3	0.8600	C8—H8	0.9300
N3—C4	1.352 (2)	C8—C9	1.368 (4)
C1—H1A	0.9700	C9—H9	0.9300
C1—H1B	0.9700	C9—C10	1.387 (3)
C1—C2	1.522 (3)	C10—H10	0.9300
C2—H2A	0.9700		
			
O1—P1—C1	106.83 (10)	H2A—C2—H2B	107.7
O2—P1—O1	104.80 (9)	N1—C3—H3A	127.2
O2—P1—C1	108.71 (11)	N1—C3—C4	105.65 (18)
O3—P1—O1	114.92 (11)	C4—C3—H3A	127.2
O3—P1—O2	110.35 (9)	N3—C4—C3	104.31 (17)
O3—P1—C1	110.89 (9)	N3—C4—C5	124.28 (17)
P1—O1—H1	109.5	C3—C4—C5	131.39 (18)
P1—O2—H2	109.5	C6—C5—C4	120.16 (19)
N2—N1—C2	118.78 (18)	C10—C5—C4	120.86 (18)
N2—N1—C3	112.94 (16)	C10—C5—C6	119.0 (2)
C3—N1—C2	128.26 (18)	C5—C6—H6	120.2
N1—N2—N3	103.64 (15)	C7—C6—C5	119.6 (2)
N2—N3—H3	123.3	C7—C6—H6	120.2
N2—N3—C4	113.45 (16)	C6—C7—H7	119.5
C4—N3—H3	123.3	C8—C7—C6	121.0 (2)
P1—C1—H1A	108.2	C8—C7—H7	119.5
P1—C1—H1B	108.2	C7—C8—H8	120.2
H1A—C1—H1B	107.4	C9—C8—C7	119.7 (2)
C2—C1—P1	116.22 (15)	C9—C8—H8	120.2
C2—C1—H1A	108.2	C8—C9—H9	119.8
C2—C1—H1B	108.2	C8—C9—C10	120.3 (2)
N1—C2—C1	113.42 (18)	C10—C9—H9	119.8
N1—C2—H2A	108.9	C5—C10—C9	120.4 (2)
N1—C2—H2B	108.9	C5—C10—H10	119.8
C1—C2—H2A	108.9	C9—C10—H10	119.8
C1—C2—H2B	108.9		
			
P1—C1—C2—N1	56.6 (3)	C2—N1—C3—C4	178.52 (19)
O1—P1—C1—C2	167.10 (17)	C3—N1—N2—N3	−0.6 (2)
O2—P1—C1—C2	54.50 (19)	C3—N1—C2—C1	65.1 (3)
O3—P1—C1—C2	−67.0 (2)	C3—C4—C5—C6	14.3 (3)
N1—N2—N3—C4	0.6 (2)	C3—C4—C5—C10	−165.7 (2)
N1—C3—C4—N3	0.0 (2)	C4—C5—C6—C7	179.59 (18)
N1—C3—C4—C5	−178.62 (18)	C4—C5—C10—C9	−178.9 (2)
N2—N1—C2—C1	−116.9 (2)	C5—C6—C7—C8	−1.1 (3)
N2—N1—C3—C4	0.4 (2)	C6—C5—C10—C9	1.0 (3)
N2—N3—C4—C3	−0.4 (2)	C6—C7—C8—C9	1.9 (4)
N2—N3—C4—C5	178.37 (17)	C7—C8—C9—C10	−1.2 (4)
N3—C4—C5—C6	−164.07 (18)	C8—C9—C10—C5	−0.2 (4)
N3—C4—C5—C10	15.9 (3)	C10—C5—C6—C7	−0.4 (3)
C2—N1—N2—N3	−178.94 (17)		

### Hydrogen-bond geometry (Å, º)

**Table:**

*D*—H···*A*	*D*—H	H···*A*	*D*···*A*	*D*—H···*A*
O1—H1···Cl1^i^	0.82	2.15	2.9521 (16)	167
O2—H2···Cl1	0.82	2.16	2.9422 (17)	160
N3—H3···O3^ii^	0.86	1.78	2.610 (2)	162
C6—H6···O2^iii^	0.93	2.58	3.476 (3)	163

Symmetry codes: (i) −*x*+2, *y*−1/2, −*z*+1/2; (ii) −*x*+1, *y*+1/2, −*z*+1/2; (iii) *x*, −*y*+3/2, *z*+1/2.
